# *O*-GlcNAcase Expression is Sensitive to Changes in *O*-GlcNAc Homeostasis

**DOI:** 10.3389/fendo.2014.00206

**Published:** 2014-12-01

**Authors:** Zhen Zhang, Ee Phie Tan, Nicole J. VandenHull, Kenneth R. Peterson, Chad Slawson

**Affiliations:** ^1^Department of Biochemistry and Molecular Biology, University of Kansas Medical Center, Kansas City, KS, USA; ^2^KUMC Cancer Center, University of Kansas Medical Center, Kansas City, KS, USA; ^3^Institute for Reproductive Health and Regenerative Medicine, University of Kansas Medical Center, Kansas City, KS, USA; ^4^KU Alzheimer’s Disease Center, University of Kansas Medical Center, Kansas City, KS, USA

**Keywords:** *O*-GlcNAc, *O*-GlcNAc transferase, *O*-GlcNAcase, post-translational modification, transcription

## Abstract

*O*-linked *N*-acetylglucosamine (*O-*GlcNAc) is a post-translational modification involving an attachment of a single β-*N-*acetylglucosamine moiety to serine or threonine residues in nuclear and cytoplasmic proteins. Cellular *O-*GlcNAc levels are regulated by two enzymes: *O*-GlcNAc transferase (OGT) and *O*-GlcNAcase (OGA), which add and remove the modification, respectively. The levels of *O*-GlcNAc can rapidly change in response to fluctuations in the extracellular environment; however, *O*-GlcNAcylation returns to a baseline level quickly after stimulus removal. This process termed *O*-GlcNAc homeostasis appears to be critical to the regulation of many cellular functions including cell cycle progress, stress response, and gene transcription. Disruptions in *O-*GlcNAc homeostasis are proposed to lead to the development of diseases, such as cancer, diabetes, and Alzheimer’s disease. *O*-GlcNAc homeostasis is correlated with the expression of OGT and OGA. We reason that alterations in *O*-GlcNAc levels affect OGA and OGT transcription. We treated several human cell lines with Thiamet-G (TMG, an OGA inhibitor) to increase overall *O-*GlcNAc levels resulting in decreased OGT protein expression and increased OGA protein expression. OGT transcript levels slightly declined with TMG treatment, but OGA transcript levels were significantly increased. Pretreating cells with protein translation inhibitor cycloheximide did not stabilize OGT or OGA protein expression in the presence of TMG; nor did TMG stabilize OGT and OGA mRNA levels when cells were treated with RNA transcription inhibitor actinomycin D. Finally, we performed RNA Polymerase II chromatin immunoprecipitation at the OGA promoter and found that RNA Pol II occupancy at the transcription start site was lower after prolonged TMG treatment. Together, these data suggest that OGA transcription was sensitive to changes in *O-*GlcNAc homeostasis and was potentially regulated by *O*-GlcNAc.

## Introduction

*O*-linked *N*-acetylglucosamine (*O*-GlcNAc) is a post-translational modification (PTM) first discovered by Gerald W. Hart and Carmen-Rosa Torres in 1984 (1). They initially used bovine milk galactosyltransferase (GalT1) to probe for terminal *N-*acetylglucosamine glycoconjugates on T-cells and unexpectedly discovered the existence of single β*-N*-acetylglucosamine conjugated proteins inside the cell (1). *O-*GlcNAc is a reversible modification that is ubiquitiously expressed in higher eukaryotes. *O-*GlcNAc transferase (OGT) is the enzyme that adds the *O-*GlcNAc modification, whereas *O-*GlcNAcase (OGA) removes it (2, 3). Because uridine diphosphate-*N-*acetyl-glucosamine (UDP-GlcNAc), the end point of the hexosamine biosynthetic pathway, is the high-energy donor substrate for OGT, *O*-GlcNAcylation is sensitive to nutrient availability (4). Furthermore, the removal and addition of *O-*GlcNAc termed *O-*GlcNAc cycling is highly dynamic. Changes in hormones, nutrients, or the environment cause within minutes to several hours changes to the total level of *O*-GlcNAc on proteins (5–7). Importantly, *O-*GlcNAc cycling rates affect transcription regulatory pathways, cell cycle progression, and respiration (8–12).

Since *O*-GlcNAcylation plays a significant role in regulating a wide panel of cellular processes, and aberrant *O-*GlcNAcylation contributes to the development of diseases, understanding the regulation of OGT and OGA is, therefore, important. Several studies report that the expression of OGT and OGA is sensitive to changes in total cellular *O-*GlcNAc levels (13, 14). Elevation of *O*-GlcNAc levels via pharmacological inhibition of OGA causes OGT protein expression to decrease and OGA protein expression to increase (13). A rapid decrease in OGA protein expression occurs in mice embryonic fibroblasts when OGT is knocked out (14). Cells appear to actively keep a specific level of *O*-GlcNAcylation suggesting a certain homeostatic level of *O*-GlcNAc must be maintained for optimal cellular function. Although alterations of cellular *O-*GlcNAc levels affect OGT and OGA expression, the exact mechanism as to explain this phenomenon is still unclear. An imbalance in the homeostasis of *O-*GlcNAc does contribute to the development of diseases including cancer, diabetes, and Alzheimer’s (15–18).

To further address how cells adjust OGT and OGA protein expression in response to alterations in *O-*GlcNAc levels, we measured in different cell lines OGT and OGA protein and mRNA expression and stability after pharmacologically inhibition of OGA by Thiamet-G (TMG, an OGA inhibitor). In these experiments, we were able to show that the OGA mRNA levels were more sensitive compared to OGT to alterations in *O*-GlcNAc, and RNA Pol II occupancy at the OGA transcription start site (TSS) was decreased after prolonged TMG treatments. Altogether, our data show that the protein expression of OGT and OGA is sensitive to changes in *O-*GlcNAc levels, and OGA transcription is sensitive to alterations in *O-*GlcNAc homeostasis.

## Materials and Methods

### Antibodies and reagents

All primary and secondary antibodies used for immunoblotting were used at a 1:1,000 and 1:10,000 dilution, respectively. Anti-O-linked *N*-acetylglucosamine antibody [RL2] (ab2739) was purchased from Abcam. Antibodies for OGT (AL-34) and OGA (345) were gracious gifts from the Laboratory of Gerald Hart in the Department of Biological Chemistry at the Johns Hopkins University School of Medicine. Actin (A2066) antibody and anti-chicken IgY HRP (A9046) were purchased from Sigma. Chromatin immunoprecipitation (ChIP) grade mouse (G3A1) mAb IgG1 isotype control (5415) and RNA polymerase II antibody, clone CTD4H8 (05-623) were purchased from Cell Signaling Technologies and Millipore, respectively. Anti-rabbit HRP (170-6515) and anti-mouse HRP (170-6516) were purchased from Bio-Rad.

All reagents were purchased form Sigma unless otherwise noted. Cycloheximide (CHX, C7698, Sigma) was used at 50 μg/ml for HeLa cells and 25 μg/ml for K562 cells (19, 20). Actinomycin D (AMD, A1410, Sigma) was used at 0.5 μg/ml for HeLa cells and 5 μg/ml for K562 cells (20, 21).

### Cell culture

HeLa cells and SH-SY5Y neuroblastoma cells were cultured in DMEM (Invitrogen) supplemented with 10% fetal bovine serum (FBS, Gemini) and 1% penicillin/streptomycin (Invitrogen). K562 cells were cultured in RPMI-1640 (Invitrogen) supplemented with 10% fetal bovine serum, 1× MEM non-essential amino acids solution (Invitrogen), 1 mM sodium pyruvate (Invitrogen), 2.5 mM HEPES, and 1% penicillin/streptomycin. Cells were treated with 10 μM Thiamet-G (TMG, S.D. Specialty Chemicals) for 6, 8, 24, or 48 h with fresh TMG supplied daily. Cells were also pretreated with CHX for 4 h, followed by TMG treatment for 8 h or AMD for 0.5 h, followed by TMG treatment for 6 h. Cells were infected with OGT, OGA, or green fluorescent protein (GFP) virus at a multiplicity of infection (MOI) of 75 for 24 h. After different treatments, cells were harvested for western blot, quantitative PCR (qPCR), or ChIP assay.

### Immunoblotting

Cells were lysed on ice for 30 min in Non-idet P-40 (NP-40) Lysis Buffer (20 mM Tris-HCl, pH 7.4, 150 mM NaCl, 1 mM EDTA, 1 mM DTT, 40 mM GlcNAc, and 1% Non-idet P-40) with 1 mM PMSF, 1 mM sodium fluoride, 1 mM β-glycerol phosphate, and 1× protease inhibitor cocktail I (leupeptin 1 mg/ml, antipain 1 mg/ml, benzamidine 10 mg/ml, and 0.1% aprotinin). Cell lysates were mixed with 4× protein solubility mixture (100 mM Tris-HCl, pH 6.8, 10 mM EDTA, 8% SDS, 50% sucrose, 5% β-mercaptoethanol, 0.08% Pyronin-Y). All electrophoresis was performed with 4–15% gradient polyacrylamide gels (Criterion Gels, Bio-Rad) and separated at 120 V, followed by transfer to PVDF membrane (Immobilon, Millipore) at 0.4 A. Blots were blocked by 3% BSA in TBST (25 mM Tris-HCl, pH 7.6, 150 mM NaCl, 0.05% Tween-20) at room temperature for 20 min, then incubated with primary antibody at 4°C overnight. After washing with TBST for 5 × 5 min, blots were incubated with HRP-conjugated secondary for 1 h at room temperature, then washed with TBST again and developed using chemiluminescent substrate (HyGlo E2400; Denville Scientific). Blots were stripped in 200 mM glycine, pH 2.5 at room temperature for 1 h and probed with different primary antibodies. All immunoblotting results were repeated in three independent experiments (9). OGA and OGT relative protein levels were measured by analyzing the bands density using ImageJ 1.48 (http://imagej.nih.gov/ij/download.html) then normalized to the density of actin. All experiments were repeated three times, and average relative fold changes were calculated.

### Total RNA isolation and RT-PCR

Total RNA was isolated by TRI reagent solution (AM9738, Ambion) according to manufacture’s instruction. Briefly, 2 × 10^6^ cells were resuspended by 1 mL TRI reagent solution. Then, 200 μl of chloroform was added to extract RNA. After spinning down, upper phase containing total RNA was collected and incubated with equal amount isopropanol. RNA pellets were then precipitated by centrifugation, washed once with 70% ethanol, air-dried, and dissolved in nuclease free water (Invitrogen).

RNA concentration was measured by Nanodrop 2000c (Thermo) and 1 μg of total RNA was used for reverse transcription (RT) using iScript Reverse Transcription Supermix (170-8841, Bio-Rad) following manufacturer’s instruction. In all, 10 μl of each completed reaction mix was incubated in a thermal cycler (Model 2720, Applied Biosystems) using the following protocol: priming 5 min at 25°C, RT 30 min at 42°C, and RT inactivation 5 min at 85°C. cDNA products were diluted with 90 μl nuclease free water and analyzed by qPCR. All qPCR results were repeated in three independent experiments (22).

### ChiP assay

K562 cells were harvested by centrifugation at 1,000 g for 5 min and washed twice with 1× PBS. Cells were then crosslinked by 2 mM EGS (21565, Pierce) in PBS at room temperature for 30 min, followed by 1% formaldehyde (BP531-25, Fisher) for another 10 min. Crosslinking reaction was terminated by 125 mM glycine. Cell pellets were collected and lysed on ice for 30 min by cell lysis buffer (10 mM Tris-HCl, pH 8.1, 10 mM NaCl, 0.5% NP-40) with protease inhibitors. Chromatin was collected by spinning down, and the pellets were resuspended in cold nuclear lysis buffer (50 mM Tris-HCl, pH 8.1, 10 mM EDTA, 1% SDS, 25% glycerol) with protease inhibitors. In total, 300 μl of nuclear lysis buffer was used to resuspend chromatin from 2 × 10^6^ cells.

Chromatin DNA was sheared to the size of 100–300 bp by sonication (Model Q800R, Active Motif) with the following protocol: amplification 75%, pulse on 15 s, pulse off 45 s, temperature 3°C. 200 μl of sheared chromatin was diluted by adding 1 ml of ChIP buffer (20 mM Tris-HCl pH8.1, 1.2% Triton X-100, 1.2 mM EDTA, 20 mM NaCl) with protease inhibitors. 2 μg of control IgG and specific antibody were added to diluted chromatin respectively, followed by end to end rotation at 4°C overnight. At the same time, 12 μl of diluted chromatin was saved as input and processed later. Next day, 30 μl of PBS washed M-280 Sheep Anti-Mouse IgG Dynabeads (11204D, Invitrogen) was added to the chromatin, followed by rotating at 4°C for 4 h. Dynabeads were separated by DynaMag-2 Magnet (12321D, Invitrogen) and subsequently washed with 1 ml of the following buffer for 5 min at 4°C: wash buffer 1 (0.1% SDS, 1% Triton X-100, 2 mM EDTA, 20 mM Tris-HCl pH 8.0, 150 mM NaCl), wash buffer 2 (0.1% SDS, 1% Triton X-100, 2 mM EDTA, 20 mM Tris-HCl pH 8.0, 300 mM NaCl), wash buffer 3 (0.1% SDS, 1% Triton X-100, 2 mM EDTA, 20 mM Tris-HCl pH 8.0, 500 mM NaCl), wash buffer 4 (0.25 M LiCl, 1% NP-40, 1% sodium deoxycholate, 1 mM EDTA, 10 mM Tris-HCl pH 8.0), and TE buffer (10 mM Tris-HCl, pH 8.0, 1 mM EDTA).

Complexes were eluted from beads with 500 μl elution buffer (1% SDS, 0.1 M NaHCO_3_, 40 mM Tris-HCl, pH 8.0, 10 mM EDTA) and added with 200 mM NaCl. Eluates and inputs were treated at the same time with RNase A (EN0531, Thermo) at 65°C overnight, followed by proteinase K (25530-031, Invitrogen) treatment for 2 h. DNA was extracted by phenol/chloroform/isoamyl alcohol (AC327111000, Fisher) and precipitated by glycogen (10814-010, Invitrogen) and ethanol (23). DNA pellets were air-dried, dissolved in 50 μl nuclease free water, and analyzed by qPCR.

### qPCR assay

cDNA or ChIP DNA samples were analyzed by qPCR using SsoAdvanced Universal SYBR Green Supermix (172-5271, Bio-Rad) according to manufacturer’s instruction. Briefly, 2 μl of cDNA or 5 μl of ChIP DNA samples, SYBR green supermix, nuclease free water, and primers (Table 1) for the target gene were mixed with a total reaction volume of 20 μl. A 96-well PCR plate (AVRT-LP, Midsci) with the mixture was loaded to CFX96 Touch Real-Time PCR Detection System (185-5195, Bio-Rad) with the following protocol: polymerase activation and DNA denaturation 30 s at 95°C, amplification denaturation 5 s at 95°C and annealing 30 s at 60 or 62°C with 40 cycles, and melt curve 65–95°C with 0.5°C increment 5 s/step.

**Table 1:** **Primer sequences used for qPCR**.

Target gene	PrP primer sequence
OGT	Forward: CATCGAGAATATCAGGCAGGAG
	Reverse: CCTTCGACACTGGAAGTGTATAG
OGA	Forward: TTCACTGAAGGCTAATGGCTCCCG
	Reverse: ATGTCACAGGCTCCGACCAAGT
HPRT	Forward: ATTGGTGGAGATGATCTCTCAACTTT
	Reverse: GCCAGTGTCAATTATATCTTCCACAA
−1000 OGA TSS	Forward: TTGGGTCTCCTTGCTGTATG
	Reverse: ACCTCACAGGTTGAGATAGATTT
OGA TSS	Forward: GGGCTAGCCTATTAAGCTTCTTTA
	Reverse: AGGGTGGCAAGCAGAAAT
+2700 OGA TSS	Forward: TCCTTTCAGAGTTGCTCCAATA
	Reverse: CAGTCAACCGAAACCATGAAC

### Data analysis

Quantification cycle (Cq) value was recorded by CFX Manager™ software. For cDNA qPCR data, dynamic range of RT and amplification efficiency was evaluated before using ΔΔCq method to calculate relative gene expression change. For ChIP DNA qPCR data, Cq value was normalized to percentage of input. Data generated in three independent experiments was presented as means ± standard error and analyzed using two-tailed Student’s *t*-test with *P* < 0.05 as significant difference.

## Results

### Alteration in *O-*GlcNAc levels changes the protein expression of OGT and OGA

**Figure 1:**
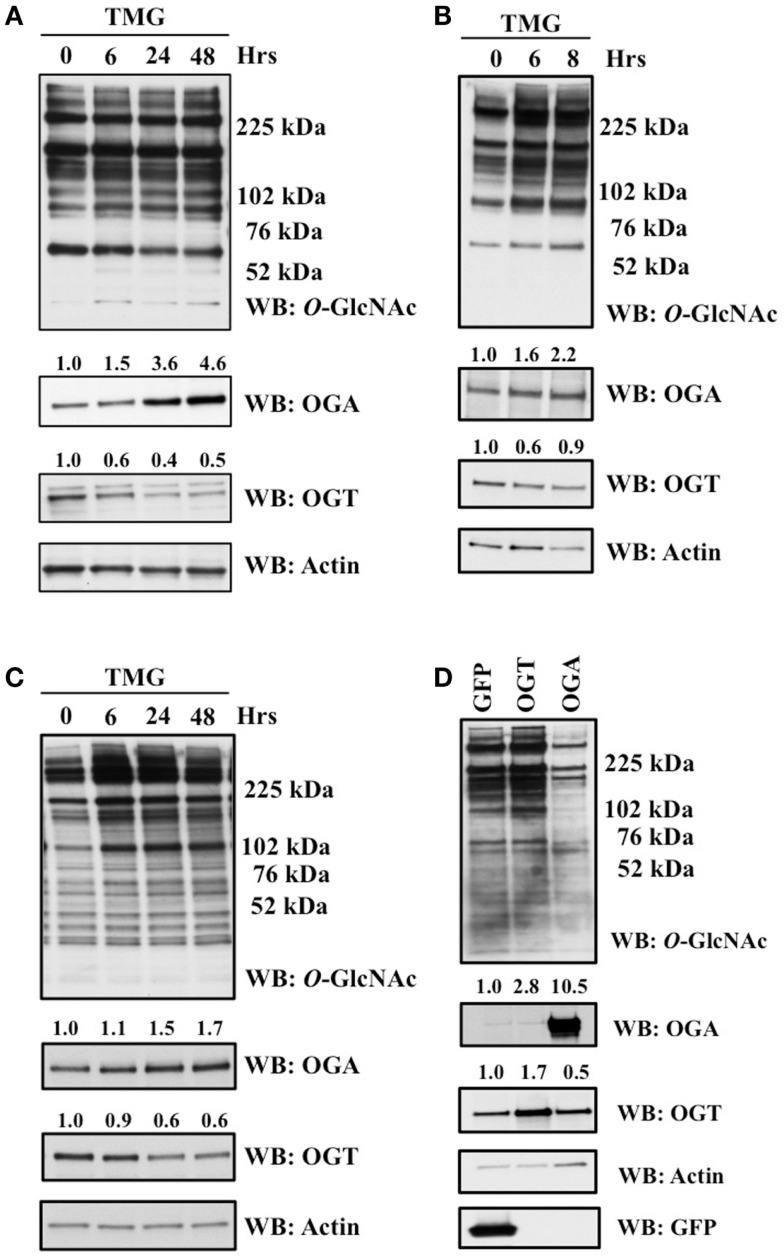
**OGA protein level was increased after TMG treatment**. **(A)** SH-SY5Y neuroblastoma cells. **(B)** HeLa cervical cells. **(C)** K562 leukemia cells were treated with 10 μM TMG for indicated time. **(D)** SH-SY5Y cells were infected with GFP, OGT, and OGA adenovirus at 75 MOI for 24 h. Cells were lysed, overall *O*-GlcNAc level, OGA and OGT protein level were analyzed by western blot with actin as a loading control. Average fold change for OGT and OGA is listed on the blots.

Previous reports demonstrated that different pharmacological inhibitors of OGA, PUGNAc and GlcNAc-thiazoline, rapidly increase the protein expression of OGA (13, 24). We explored this phenomenon using another highly selective inhibitor of OGA Thiamet-G (TMG) (25). We altered the *O-*GlcNAc levels of SH-SY5Y neuroblastoma, HeLa cervical carcinoma, and K562 leukemia cells with TMG and measured *O*-GlcNAc, OGT, and OGA levels at various time points up to 48 h of TMG treatment. The *O-*GlcNAc levels were increased in the TMG treated samples while the pattern of *O-*GlcNAcylation was unique to each of the three cells lines used. OGA protein expression increased while OGT protein expression decreased gradually in the prolonged TMG treatment time points (Figures 1A–C). Additionally, in SY5Y cells, we used adenoviral-mediated OGT or OGA infection to alter *O*-GlcNAc levels. GFP was used as a control for the adenoviral infection. Cells overexpressing OGT showed an elevation in *O-*GlcNAc levels and a slight increase in OGA protein expression compared to control, while cells overexpressing OGA showed a decrease in *O-*GlcNAc levels and a slight increase in OGT protein expression compared to control (Figure 1D).

### TMG does not stabilize OGA protein expression

**Figure 2:**
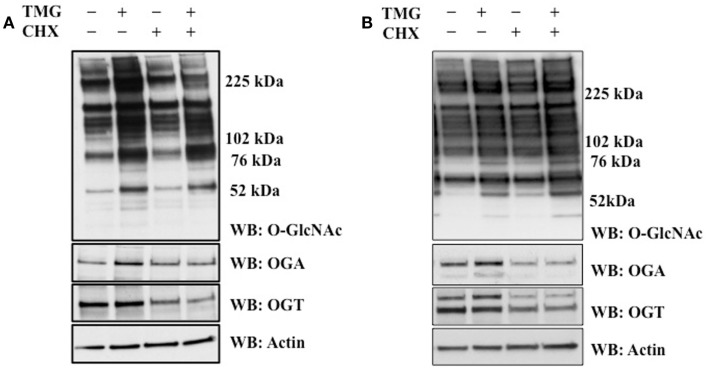
**TMG does not stabilize OGA protein**. **(A)** HeLa cells and **(B)** K562 cells were treated with TMG, CHX (protein translation inhibitor), and CHX + TMG. Cells were lysed, overall *O*-GlcNAc level, OGA and OGT protein level were analyzed by western blot, with actin as loading control.

In order to explore the reason why TMG increases OGA protein level, we asked the question does TMG increase OGA protein stability. We pretreated cells with CHX to inhibit protein translation (26). We treated HeLa (Figure 2A) and K562 (Figure 2B) cells with TMG and observed a robust increase in OGA protein level (Figures 2A,B, Lane 2) compared to control cells without any treatment (Figures 2A,B, Lane 1). When HeLa cells were treated with CHX, OGT protein levels dramatically decreased compared to control, and we did not observe much of a decrease in OGA protein expression (Figure 2A, Lane 3). However, both OGT and OGA protein levels were dramatically decreased after CHX treatment in K562 cells (Figure 2B, Lane 3) compared to control. Combination of CHX and TMG did not change the OGA or OGT protein levels (Figures 2A,B, Lane 4) compared to CHX treatment only suggesting that the TMG mediated increase in OGA protein expression was not due to increased stability of the protein.

### OGA transcript level is increased after TMG treatment

**Figure 3:**
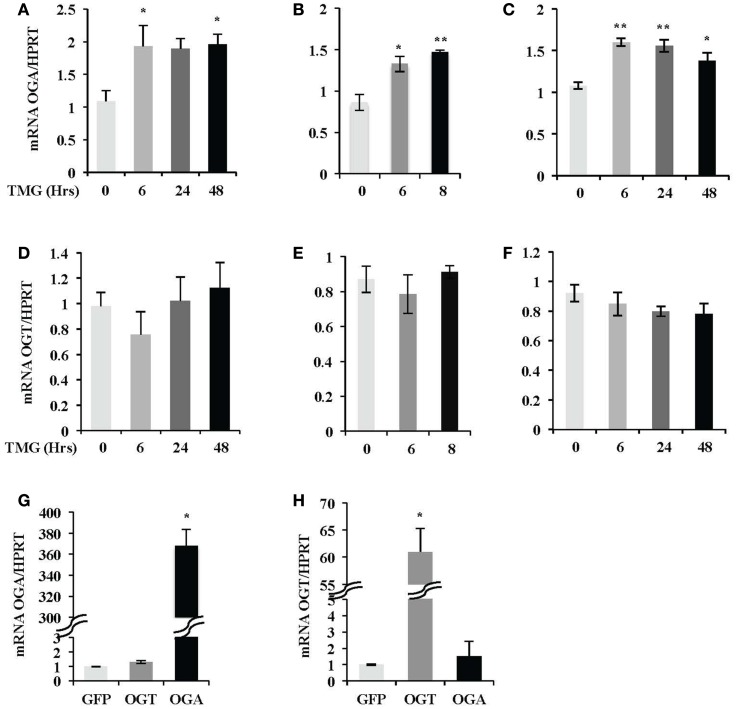
**OGA mRNA level was increased after TMG treatment**. After TMG treatment, relative OGA mRNA level in **(A)** SH-SY5Y, **(B)** HeLa, and **(C)** K562 cells, as well as OGT mRNA level in **(D)** SH-SY5Y, **(E)** HeLa, and **(F)** K562 cells was analyzed by qPCR. **(G)** OGA mRNA level and **(H)** OGT mRNA level in SH-SY5Y cells infected with GFP, OGT, and OGA adenovirus at 75 MOI for 24 h, respectively, were also analyzed by qPCR. Hypoxanthine- guanine phosphoribosyltransferase (HPRT) was served as internal control. *P < 0.05. **P < 0.01, compared with control (TMG 0 h or GFP), *n* = 3, Student’s *t*-test.

Next, we investigated if OGT or OGA transcript level was altered after TMG treatment. We analyzed OGA mRNA level in SH-SY5Y (Figure 3A), HeLa (Figure 3B), and K562 (Figure 3C) cells. We found OGA mRNA level increased from 6 h TMG treatment in all three cell lines and was still elevated above control after 48 h TMG treatment (Figures 3A,C). The OGA mRNA level corresponded with the increase in protein level in Figure 1. However, the OGT mRNA level did not significantly change (Figures 3D–F). We also demonstrated that the corresponding OGA and OGT mRNA levels increased slightly but not significantly when OGT or OGA were overexpressed in SH-SY5Y cells (Figures 3G,H).

### TMG does not stabilize OGA mRNA

**Figure 4:**
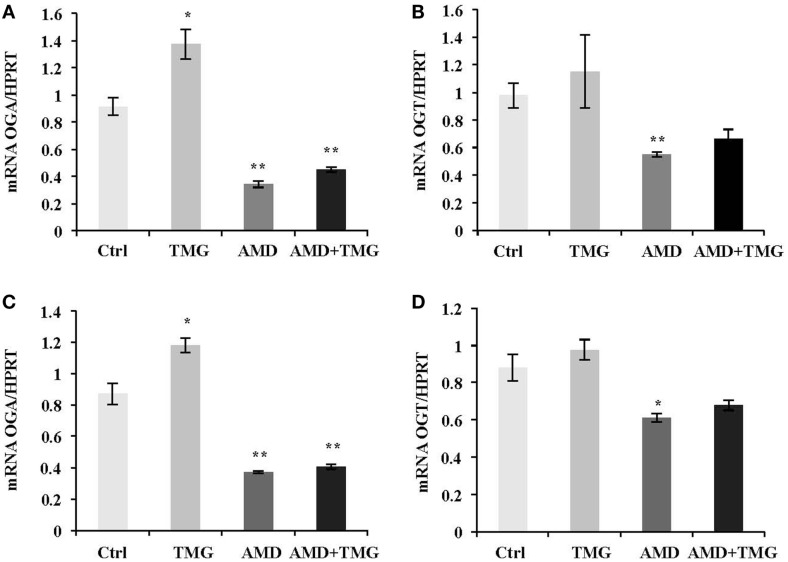
**TMG does not stabilize OGA mRNA**. HeLa cells **(A,B)** and K562 cells **(C,D)** were treated with TMG, AMD (RNA transcription inhibitor), and AMD + TMG, respectively. **(A)** OGA **(A,C)** and OGT **(B,D)** mRNA level were analyzed by qPCR, with HPRT as internal control. *P < 0.05. **P < 0.01, compared with control, *n* = 3, Student’s *t*-test.

Next, we asked the question whether increased OGA mRNA level after TMG treatment was due to stabilized OGA mRNA. AMD, a RNA synthesis inhibitor, was used to test OGA mRNA stability. TMG treated HeLa cells showed an increase of OGA mRNA level compared to control cells without any treatment (Figure 4A). When cells were treated with AMD, both OGA and OGT mRNA levels were dramatically decreased compared to control (Figures 4A,B). Combination of AMD and TMG did not change the OGA and OGT mRNA levels compared to AMD treatment only (Figures 4A,B). The same results were observed when using K562 cells (Figures 4C,D).

### RNA Pol II occupancy is decreased at OGA TSS after 48 h TMG treatment

**Figure 5:**
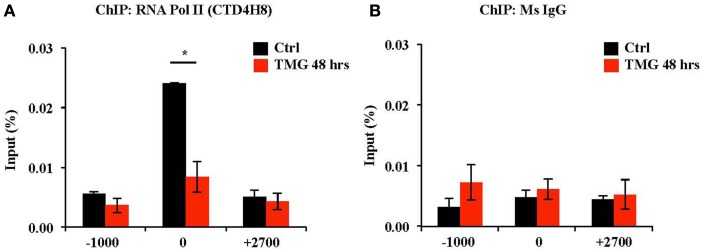
**RNA Pol II occupancy at OGA TSS was decreased after 48 h TMG treatment in K562 cells**. **(A)** RNA Pol II ChIP assay was performed on control and 48 h TMG treated cells. ChIP DNA was analyzed by qPCR using a set of primer targeting 1000 bp upstream of OGA TSS (−1000), OGA TSS (0), and +700 bp downstream of OGA TSS (+2700). *P < 0.05, *n* = 3, Student’s *t*-test. **(B)** Normal mouse IgG ChIP served as a negative control.

We next investigated RNA Pol II occupancy at the OGA TSS via RNA Pol II ChIP. In control K562 cells, RNA Pol II was bound to OGA TSS with little binding upstream (−1000) or downstream (+2700) of the TSS. However, after 48 h TMG treatment, RNA Pol II occupancy was decreased at the OGA TSS compared to control cells (Figure 5A). Normal mouse IgG ChIP was used as a negative control (Figure 5B).

## Discussion

The production of UDP-GlcNAc, the substrate for OGT, integrates various metabolic substrates allowing the *O*-GlcNAc modification to act as a nutrient sensor (4, 27). Consequently, cells are sensitive to changes in *O*-GlcNAc levels due to nutritional and metabolic flux and will adjust cellular functions accordingly. Prolonged alterations in homeostatic levels of *O*-GlcNAc will cause the protein expression of OGT and OGA to change in an effort to restore *O*-GlcNAc homeostasis (4). Exactly how cells sense alterations to homeostatic levels of *O*-GlcNAc and adjust OGT and OGA expression to compensate for the changes in *O*-GlcNAcylation is unclear. For example, pharmacological inhibition of OGA rapidly increases cellular *O*-GlcNAc levels; however, the protein expression of OGA will also increase in response to the elevation of *O*-GlcNAc (13, 24). We sought to explore the mechanism as to how OGT and OGA protein expression changes in response to alterations in cellular *O*-GlcNAc levels. In agreement with previous reports, we found an increase in OGA protein expression as quickly as 8 h in HeLa cells and 24 h in K562 and SY5Y cells after treatment with TMG. OGT protein expression also decreased in these later time points (Figure 1). Due to the fact that increased levels of *O*-GlcNAc can increase the stability of proteins, such as p53 (28) and TET (ten-eleven translocation) (29), we postulated that increased *O*-GlcNAc could stabilize OGA. K562 or HeLa cells exposed to CHX in the presence of TMG showed no difference in the stability of either OGT or OGA (Figure 2) suggesting that the increase in OGA protein expression was not due to increased stability and more likely to an increase of OGA transcripts.

Importantly, decreased *O-*GlcNAc levels do not necessarily increase OGT levels in all cell types; for example, blocking GFAT (glutamine fructose-6-phosphate amidotransferase) activity with 6-diazo-5-oxo-l-norleucine (DON) in HeLa cells lowered *O*-GlcNAc levels but did not increase OGT protein expression (13). On the other hand, OGA protein levels quickly decreased after *Cre*-mediated knockout of OGT in mouse embryonic fibroblasts (14), but OGA knockdown in colon cancer cells did not significantly decrease OGT protein expression (30). Changes in OGA protein expression appear more sensitive to changes in *O*-GlcNAc than OGT in HeLa cells, while both OGT and OGA expression significantly changed in SY5Y cells (Figure 1). Overexpression of OGA did not substantially influence OGT protein expression (Figure 1), and OGT overexpression did not change OGA expression (Figure 1). Recently, the development of a selective OGT inhibitor allowed for a dramatic reduction in cellular *O-*GlcNAcylation (31), which in turn caused OGA protein expression to rapidly decrease with only a minimal increase in OGT protein expression (31). The dynamic change in OGA protein expression was seen in the development of disease as well. In red blood cells of prediabetic individuals, OGA expression was significantly increased (32), and OGA protein levels correlated with increased blood glucose in these prediabetic patients. These data suggest that higher blood glucose levels promote increased flux through the hexosamine biosynthetic pathway leading to elevated OGT activity, followed by OGA protein levels increasing to restore cellular *O*-GlcNAc homeostasis in erythrocyte precursor cells. Together, these data support the proposed hypothesis that if OGT acts as a nutrient sensor allowing for rapid changes in *O-*GlcNAcylation due to alterations, the cellular concentration of UDP-GlcNAc (33), then OGA should be less sensitive to nutrient changes and more sensitive to changes in *O-*GlcNAcylation.

In order to respond to changes in *O*-GlcNAc levels, cells rapidly and dramatically alter the expression of OGA mRNA (Figure 3). In the case of OGT, we did not detect a significant change in OGT mRNA levels after TMG treatment. The rapid increase in OGA mRNA levels after TMG treatment would argue that either OGA transcripts were more stable or transcriptional activity at the OGA promoter was increasing. We tested transcript stability by inhibiting RNA polymerase II with AMD (21). Interestingly, OGA and OGT transcript levels were not more stable after TMG treatment in the presence of AMD (Figure 4) suggesting that the increase in OGA mRNA levels with TMG was due to an increase in OGA gene transcription.

Next, we performed ChIP at the OGA promoter with an antibody that recognized all forms of RNA Pol II (phosphorylated and non-phosphorylated forms). After 48 h of prolonged TMG treatment in K562 cells, total RNA Pol II at the promoter was decreased compared to the control samples (Figure 5). Potentially, an antibody directed against the actively transcribing phosphorylated C-terminal domain (CTD) of RNA Pol II might have demonstrated an increase in enrichment of the phosphorylated forms of RNA Pol II at the promoter while non-phosphorylated forms of RNA Pol II would be less associated with the promoter. Interestingly, RNA Pol II is *O-*GlcNAcylated on the CTD at the fourth position of the CTD repeat, which is between the two activating phosphorylations at serine two and serine five on the CTD, which is needed for transcriptional elongation (34). Both *O*-GlcNAcylation and phosphorylation appeared to be mutually exclusive suggesting a cycle of *O*-GlcNAcylation and phosphorylation on the CTD repeats (35). Several groups have suggested that OGT and OGA work together to promote gene transcription by organizing the RNA Pol II preinitiation complex (PIC) (11, 36). *O*-GlcNAcylation was shown to promote the formation of the PIC in an *in vitro* transcription assay system; however, OGA activity was required for full transcriptional activation suggesting that OGT modified RNA Pol II, which initiated the formation of the PIC, while OGA was then required to remove the *O*-GlcNAc on the stalled RNA Pol II allowing for phosphorylation and transcription elongation (11). We have yet to explore RNA Pol II occupancy at the OGA promoter after a short TMG treatment (for example 6 h), which might yield a different result and needs to be studied further. The mRNA levels of OGA in K562 cells did begin to decrease at the 48 h TMG treatment suggesting that the OGA promoter might become inactive after prolonged TMG treatment. Reciprocal binding of OGT and OGA at active gene promoters provides several interesting future questions into the nature of transcriptional regulation, and the control of both the OGT and OGA promoter might be regulated in this manner.

Many transcription factors are modified by *O-*GlcNAc (15) and likely alteration of the *O-*GlcNAcylation level of a transcription factor could mediate the change in OGA transcription. We used the predictive software TFSEARCH (http://www.cbrc.jp/research/db/TFSEARCH.html) to identify potential transcription factor-binding sites in the first 1000 base pairs upstream of the OGA TSS (37). Among the transcription factor-binding sites in this sequence, GATA and MZF were the most predicted transcription factors. Due to the essential and ubiquitous expression of OGA (4), we anticipated that several housekeeping transcription factors might bind to this region, but we found only few of these. Interestingly, both GATA and MZF are important transcription factors regulating hemopoietic development (22, 38). Perhaps the increased in OGA expression in prediabetic red blood cells (32) was partially due to changes in either of these two proteins. *O-*GlcNAcylation changes might lead to alteration of GATA or MZF occupancy at the OGA promoter. Some GATA family members are modified by *O*-GlcNAc (39); thus, this presents an interesting avenue to explore in more detail.

Together, our data demonstrate that OGA protein and mRNA expression is sensitive to cellular levels of *O*-GlcNAc. Some disease states have OGA expression uncoupled from *O*-GlcNAc levels (40). In many different cancers, *O*-GlcNAc homeostasis appears to be disrupted with increased OGT protein expression and *O*-GlcNAc levels (41). Several pancreatic cancer cell lines have increased *O*-GlcNAc levels when compared to an immortalized control cell line; importantly, OGT protein expression was increased while OGA protein expression was decreased (40). The uncoupling of OGA expression to *O*-GlcNAc homeostasis could be an indicator of cancer progression and suggest that an increase of OGA protein expression would be beneficial therapeutically. Determining how *O*-GlcNAc regulates OGA expression and transcription will be crucial for understanding the biology of *O*-GlcNAcylation and how *O*-GlcNAc homeostasis is disrupted in disease.

## Conflict of Interest Statement

The authors declare that the research was conducted in the absence of any commercial or financial relationships that could be construed as a potential conflict of interest.
